# Effect of Thermal Ageing on the Mechanical Strength of Carbon Fibre Reinforced Epoxy Composites

**DOI:** 10.3390/polym13122006

**Published:** 2021-06-19

**Authors:** Nicola Zavatta, Francesco Rondina, Maria Pia Falaschetti, Lorenzo Donati

**Affiliations:** Department of Industrial Engineering DIN, University of Bologna, Via Fontanelle 40, 47121 Forlì, Italy; nicola.zavatta2@unibo.it (N.Z.); francesco.rondina2@unibo.it (F.R.); l.donati@unibo.it (L.D.)

**Keywords:** thermo-oxidative, temperature, mechanical properties, carbon fabric, CFRP, thermoset

## Abstract

Applications of Carbon Fibre Reinforced Polymers (CFRP) at temperatures over 150–200 °C are becoming common in aerospace and automotive applications. Exposure of CFRP to these temperatures can lead to permanent changes in their mechanical properties. In this work, we investigated the effect of thermal ageing in air on the strength of carbon fabric/epoxy composites. To this end, accelerated artificial ageing at different temperatures was performed on carbon fabric/epoxy specimens. The flexural and interlaminar shear strengths of the aged specimens were assessed by three-point bending and short beam shear tests, respectively, and compared to those of unaged samples. For ageing at temperatures below the glass transition temperature of the resin, $T_{g}$, a moderate reduction of strength was found, with a maximum decrease of 25% for 2160 h at 75% $T_{g}$. On the other hand, a rapid strength decrease was observed for ageing temperatures above $T_{g}$. This was attributed to degradation of the epoxy matrix and of the fibre/epoxy interface. In particular, a 30% strength decrease was found for less than 6 h at 145% $T_{g}$. Therefore, it was concluded that even a short exposure to operating temperatures above $T_{g}$ could substantially impair the load-carrying capability of CFRP components.

## 1. Introduction

Composite materials have been widely adopted in a number of applications in the automotive and aerospace industry. Due to their high strength-to-weight ratio, Carbon Fibre Reinforced Polymers (CFRP) are particularly attractive for lightweight components such as aircraft panels, car parts, and cables [1,2,3,4,5]. Among them, carbon/epoxy composites stand out for their excellent mechanical properties. One of their main drawbacks is their decreasing mechanical performance with temperature increase which, combined with their low thermal conductivity, makes them much more susceptible to heating than traditional metallic materials. While the use of CFRP in thermal-critical applications was generally avoided in the past, the request for composites in lightweight applications is pushing their use to more demanding conditions. In particular, in some components (like wheels, parts of combustion engines, or parts located close to exhaust pipes), operating temperatures as high as 150–300 °C can occur as a result of heating by nearby heat sources. Therefore, thermal ageing caused by exposure to such temperatures can become a major issue over the component’s lifetime. Accurate assessment of the mechanical properties of the material aged under these conditions is of paramount importance for the design of composite structures [6,7,8].

For temperatures below 400 °C, the effect of carbon fibres oxidation is negligible [9,10,11]. Consequently, in the range 150–300 °C the response of fibre/epoxy composites to ageing is dominated by the response of the neat resin, as observed in a comprehensive review by Odegard and Bandyopadhyay [12]. The effect of thermal ageing on the mechanical properties of pure epoxy resins has long been studied [13,14,15,16]. Merrall and Meeks [13] reported an initial increase of the tensile strength and modulus for isothermal ageing. After this initial increase, both quantities dropped. The decrease in strength and modulus was much more marked for temperatures above the glass transition temperature of the epoxy, $T_{g}$. On the other hand, an increase in both tensile strength and modulus was found by Silva et al. [15] for non-isothermal ageing at temperatures greater than $T_{g}$. Similarly, Qin et al. [16] observed an improvement in the tensile strength of Araldite resin subjected to isothermal ageing at a temperature above $T_{g}$. Basically, no change of the flexural modulus was reported in [14] for sub-$T_{g}$ ageing in vacuum. The strength increase due to thermal ageing observed in [13,15,16] can be explained as a result of resin post-curing promoted by temperature. Generally, ageing was found to result in weight loss, which has been attributed to chemical degradation of the epoxy [17].

Several works have explored the effect of thermal ageing on carbon fibre/epoxy composites. Although some of them address the combined effect of temperature and other factors, such as moisture, i.e., hygrothermal ageing [18], moisture and pressure [19], moisture, pressure and fatigue loads [20], or other sources such as UV radiation [21], in the following, we will consider solely the articles concerned with the influence of ageing temperature and time. Tsotsis [22] investigated the effect of ageing on unidirectional compression, open-hole compression, and fracture properties of CFRP, using two different epoxies. The mechanical properties of both materials were found to worsen for temperatures near $T_{g}$. A subsequent work by Tsotsis et al. [23] employed different carbon fibre/epoxy systems, which were aged at a lower temperature and different air pressures. For the specimens aged at atmospheric pressure, only a slight decrease of the tensile shear strength was observed. García-Moreno et al. [24,25] studied the effect of ageing on the impact and flexural behaviour of CFRP. Notably, they found an increase in the flexural strength of specimens aged at a temperature lower than $T_{g}$, whereas a clear deterioration was observed for higher temperatures. A significant decrease of the compressive strength caused by ageing temperatures near $T_{g}$ has also been reported by Zhang et al. [26] for 3D braided carbon fibre/epoxy specimens, while Fiamegkou et al. [27] found a reduction of the interlaminar shear strength in carbon fabric/epoxy laminates.

The aforementioned works evidence that exposure to temperatures close to the glass transition temperature of the material can have a substantial effect on the mechanical properties of CFRP. To the best of our knowledge, however, information on how different ageing conditions, i.e., different temperatures and durations, affect the strength of carbon fabric/epoxy composites is still limited.

In this work, we aim to investigate the effect of different thermal ageing conditions on the strength of a carbon fabric composite made with an epoxy resin developed for automotive and aerospace applications. To this end, we performed accelerated ageing on carbon fabric/epoxy specimens, using different combinations of temperatures (in the range 90–260 °C) and exposure times (0–2160 h). The range of temperatures was selected according to estimated ageing conditions of thermal-critical components identified in automotive and aerospace applications. In the analysis, the temperatures were then related to the initial $T_{g}$ in order to provide a more general discussion. While exposure to constant temperatures is not common in real components, isothermal ageing can be conveniently used for accelerated ageing tests. Moreover, it allows a straightforward comparison of the effects of different temperatures and durations by limiting the factors under investigation (i.e., neglecting parameters of cyclic thermal ageing like heating and cooling rates, holding times at elevated and room temperatures, etc.). The flexural and interlaminar shear strengths of the aged specimens were assessed by three-point bending and short beam shear tests, respectively. Unaged samples were also tested and used as a baseline. Additional specimens were aged under non-isothermal conditions by exposing them to low/high temperature cycles. For this group, the same tests were conducted and their results compared to those of the isothermally aged specimens, to investigate the effect of icing/deicing cycles on the mechanical strength.

## 2. Materials and Methods

All the specimens were produced from a single plate, which consisted of 4 pre-preg plies. Commercial CC 600 ER450 12KT700 2/2 Twill Carbon Fabric pre-preg plies were used, which consist of T700 fibres woven into a twill fabric and impregnated with SAATI ER450 epoxy resin. ER450 is a toughened epoxy resin developed specifically for a wide range of structural applications in the automotive and aerospace industry [28]. The characteristics of the pre-preg are reported in Table 1. The stacking sequence of the laminate was ${\left[\left(0/90\right)f/\left(90/0\right)f\right]}_{s}$. Curing was carried out in an autoclave at 135 °C and 4 bar for 2 h, according to the instructions reported in the material datasheet. The laminate was then subjected to post-curing in air at a temperature of 200 °C for 2 h and cut into smaller samples with a size of 120.0 mm × 19.0 mm × 2.6 mm. All samples were weighed on a digital scale to establish a baseline for subsequent comparison.

Prior to ageing, the glass transition temperature of the material was measured by Dynamic Mechanical Thermal Analysis (DMTA) using a Rheometric Scientific DMTA V3E analyser. The tests were conducted on four flat rectangular samples which were cut from the plate described above. A single cantilever configuration was used in the tests, with an oscillating frequency of 1 Hz and a heating rate of 2 °C/min. Two methods were used to assess the glass transition temperature $T_{g}$, as described in ASTM D7028 [30]. In the first one, $T_{g}$ was found by intersecting two tangent lines on a plot of the storage modulus versus temperature. In the second method, $T_{g}$ was identified as the peak temperature of the tangent delta curve. The two methods resulted in a $T_{g}$ of 180 and 203 °C, respectively. Since the first value, i.e., 180 °C, corresponds to the point where the mechanical properties begin to drop, this was chosen as the reference glass transition temperature for thermal ageing. In comparison, the resin datasheet reports a value of $T_{g}$ (computed according to the first method) of 167 °C for curing at 135 °C for 2 h followed by post-curing at 200 °C for 1 h, and one of 180 °C for curing at 130 °C for 1.5 h followed by post-curing at 180 °C for 2 h.

The samples were subjected to isothermal ageing under air exposure in ovens for different temperatures and durations. The temperature in each oven was measured by means of a thermocouple. The maximum temperature tested was 260 °C, which was deemed representative of real automotive applications. Between room temperature and this value, four other temperatures were selected, as detailed in Table 2. For each temperature, different durations up to a maximum of three months were explored. Samples aged at room temperature were also tested and used as a baseline.

In addition to the isothermally aged samples, other samples were subjected to cyclic ageing, i.e., cyclic variation of temperature, to assess whether alternating icing/deicing cycles could promote a further strength reduction compared to isothermal ageing. Each cycle consisted in applying a temperature of −20 °C for 12 h, followed by a temperature of 90 °C for another 12 h. These thermal cycles were repeated until the total ageing time was reached. The same ageing times that had been considered for isothermal ageing at 90 °C (50% $T_{g}$) were used, namely 6, 24, 72, 168, 360, 720, and 2160 h. For the 6-h ageing, the duration of each half-cycle was shortened to 3 h. This ageing condition is representative of an operating environment in which the component is subjected to alternating temperatures, which result in repeated freezing/thawing cycles, like repeated car braking in a below-zero environment or airplane take-off and landing from equatorial countries.

Every sample was weighed again after ageing to assess the weight loss due to thermal ageing. The percent weight loss was computed according to the following formula:

(1) $$\%\;w=\frac{w_{i}-w_{f}}{w_{i}}\times 100$$

where $w_{i}$ and $w_{f}$ are the sample’s weights before and after ageing, respectively.

In order to assess the effect of thermal ageing on the mechanical strength of the laminate in the selected range of temperatures, flexural and interlaminar shear tests were selected, because only a reduction of the matrix-based properties was expected [9,10,11]. Every sample was cut into two smaller specimens, one to be used for three-point bending tests and the other for short beam shear tests. The specimens used for the three-point bending tests had a nominal size of 100.0 mm× 19.0 mm × 2.6 mm, while those employed for the short beam shear tests had a size of 20.0 mm × 19.0 mm × 2.6 mm. For each test type and ageing condition, five specimens were tested.

The three-point bending tests were conducted according to the EN ISO 14125 standard [31]. The tests were run under displacement control with a displacement rate of 3 mm/min. The flexural strength was computed as:

(2) $$\sigma_{f}=\frac{3FL}{2bh^{2}}$$

where F is the force at failure measured by the test machine, while L, b, and h are the specimen span, width, and thickness, respectively. The short beam shear tests were conducted according to the EN ISO 14130 standard [32]. The tests were run under displacement control with a displacement rate of 0.6 mm/min. The interlaminar shear strength was calculated by means of the following expression:

(3) $$\tau_{f}=\frac{3F}{4bh}$$

where F is the force at failure measured by the test machine and b and h are specimen width and thickness.

## 3. Results and Discussion

### 3.1. Weight Loss

**Figure 1:**
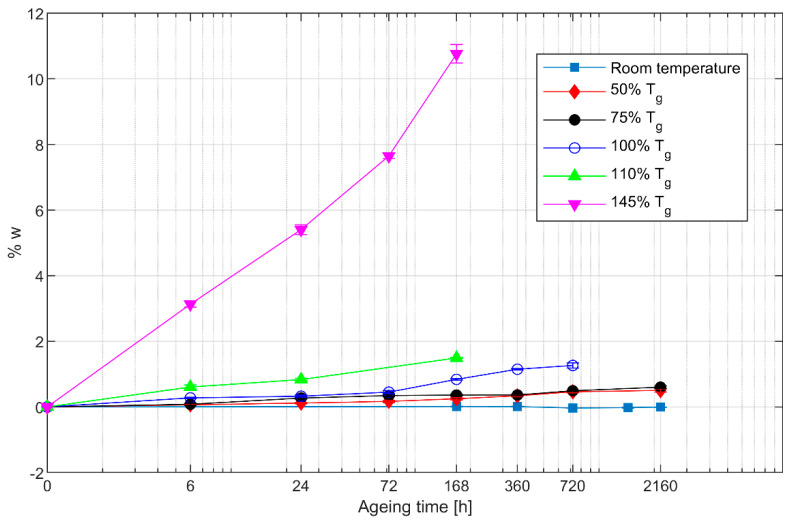
Results of weight loss measurements divided according to ageing temperature, expressed as a percentage of the glass transition temperature. The logarithmic scale on the horizontal axis has been adjusted so that the leftmost point represents the specimens in the unaged condition.

The weight loss due to thermal ageing was measured by weighing each sample before and after ageing. The resulting percent weight losses are reported in Figure 1. For each ageing condition, the mean value and standard deviation are shown on the graph.

The baseline results show that the weight remains constant at room temperature (approximately 20 °C). On the contrary, a clear effect of thermal ageing on weight loss is visible for higher temperatures. This effect gets more pronounced as the temperature increases and the weight loss becomes especially significant for ageing above the glass transition temperature. While 2160 h at 50% $T_{g}$ produce only a 0.5% weight loss, a temperature equal to $145\%\;T_{g}$ applied for 6 h causes a weight loss of more than 3%.

The significant weight loss observed here has been reported previously for neat epoxy [13,17] and has been associated with a reduction of the mechanical properties of the resin. Tsotsis [22] found a weight loss as high as 1.2% in carbon fibre/epoxy specimens aged for 1000 h at a temperature roughly equal to the glass transition temperature of the resin, which is similar to the result we found for 720 h at 100% $T_{g}$.

The rapid weight loss observed for temperatures above $T_{g}$ is an indication of material degradation, which could result in a reduction of strength. Although the exact mechanism behind the weight loss is not entirely clear, it is believed to be caused principally by structural changes in the epoxy resin. In fact, previous works by Li and Xian [9,10,11] have shown that carbon fibres are mostly unaffected by temperatures below 400 °C. Since the present experiments were conducted in air, a mechanism similar to that suggested by Buch and Shanahan [17], involving both purely thermal degradation, i.e., scission of the polymer chains, and thermal oxidation of the epoxy is deemed responsible for weight loss.

### 3.2. Three-Point Bending Tests

**Figure 2:**
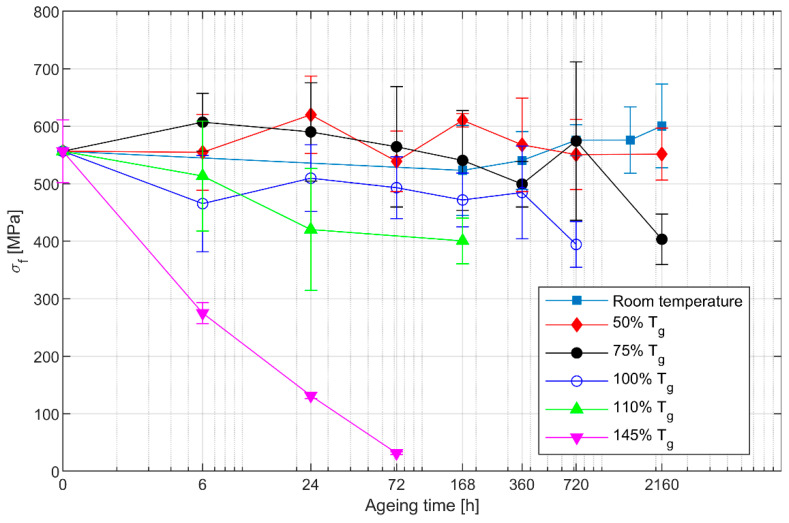
Flexural strength of the isothermally aged specimens. The logarithmic scale on the horizontal axis has been adjusted so that the leftmost point represents the specimens in the unaged condition.

The influence of ageing on the flexural properties of the laminate was assessed by three-point bending tests. For each ageing condition, five specimens were tested. In all samples, failure was initiated by compression in the region close to the loading nose. In the specimens aged at 145% $T_{g}$ for 168 h, severe deterioration was observed, so they were excluded from the tests. The flexural strength measured for the different ageing conditions is reported in Figure 2.

The response of flexural strength to ageing was found to depend largely on the ageing temperature. For ageing temperatures, lower than the glass transition temperature, namely 50% $T_{g}$ and 75% $T_{g}$, the flexural strength was found to initially increase. After this initial phase, the strength decreased to its original value. Only after 2160 h at 75% $T_{g}$ was a significant reduction of the flexural strength measured. On the other hand, for temperatures greater than the glass transition temperature, a rapid decrease of the flexural strength was observed.

**Figure 3:**
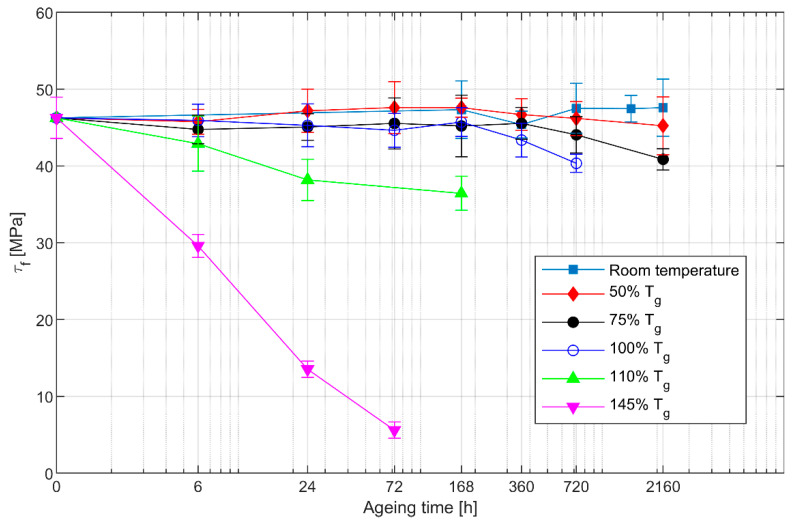
Interlaminar shear strength of the isothermally aged specimens. The logarithmic scale on the horizontal axis has been adjusted so that the leftmost point represents the specimens in the unaged condition.

With the exception of ageing at 145% $T_{g}$, the data exhibit a significant scatter, particularly when compared to those of interlaminar shear strength (Figure 3). This could be thought to be a consequence of the complex fracture mechanism of the flexural specimens. In fact, while the interlaminar shear tests are mainly dominated by matrix fracture, flexural failure results from a combination of fibre and matrix fracture. How thermal ageing affects this failure mechanism is still unclear, but it seems likely that this is responsible for the large scatter observed in the results. For ageing at 145% $T_{g}$, it is plausible that the matrix is so deteriorated that in all specimens, failure occurs for similar values of the external load, which would explain the small scatter observed.

The strength increase for lower ageing temperatures can be explained by further curing of the resin, which adds to the post-curing applied after manufacturing. This effect has been reported earlier by other authors for both neat epoxy and fibre/epoxy composites [13,15,16,25,26]. On the other hand, exposure to higher temperatures results in epoxy deterioration, which leads to a reduction of flexural strength. As noted earlier, for the temperatures considered here, the fibres remain unaffected. A significant decrease of flexural strength for temperatures above $T_{g}$ has also been observed by García-Moreno et al. [25], which is similar to that found here.

In general, it seems that, while even prolonged exposure to temperatures below $T_{g}$ causes only a moderate reduction of strength, once the ageing temperature exceeds the glass transition temperature, any further temperature increase results in a rapid strength decrease. For instance, let us consider the time required for flexural strength to decrease by 30% from its initial value of 560 MPa: for ageing at 50% $T_{g},$ the reduction of strength after three months is still negligible; at 75% $T_{g}$, the 30% decrease is reached after 2160 h and at 100% $T_{g}$ after 720 h; for 110% $T_{g},$ the necessary exposure time reduces to 168 h, while for a temperature equal to 145% $T_{g},$ it is less than 3 h. This example has a clear implication on design of composite structures: should a component be exposed to unexpected heating, even short periods above $T_{g}$ could be enough to substantially impair its load-carrying capability. Great care should therefore be taken when designing CFRP components placed near potential heat sources, such as engines, hot air ducts, or solar panels.

### 3.3. Short Beam Shear Tests

Since ageing was expected to affect mostly the properties of the epoxy matrix, short beam shear tests were conducted to assess the resistance of the laminate to interlaminar shear loads. The results of the tests are shown in Figure 3. Each point on the graph represents the mean value and standard deviation of the five specimens tested for that specific ageing condition.

The interlaminar strength results follow the same trend observed for the flexural strength, evidencing a clear decrease of the shear strength for higher ageing temperatures. In particular, a rapid deterioration of the interlaminar strength is apparent for temperatures above $T_{g}$. The specimens aged for 168 h at 145% $T_{g}$ presented severe delamination and were not included in the tests. Ageing at 50% $T_{g}$ resulted in an initial increase of the shear strength, similarly to what was found for the flexural strength. This initial increase was then followed by a strength drop for longer ageing times. It should be noted that for this ageing temperature, the scatter in the data is such that the decrease of shear strength cannot be assessed definitively. For temperatures equal to 75% $T_{g}$ and above, the decrease of interlaminar strength becomes more and more evident and, at 145% $T_{g}$, a 6-h ageing time results in a strength that is 2/3 of its original value.

That thermal ageing could reduce the shear strength of CFRP has also been reported by Fiamegkou et al. [27]. In that work, two different epoxies were considered, while the ageing consisted in a temperature equal to 230 °C, which corresponds roughly to 95% $T_{g}$, applied for a time of 720 h. The authors reported a reduction of shear strength of about 20% and 30% for the two resins considered. Compared to these figures, a slightly lesser decrease was observed in our tests, with a strength reduction of about 14% for ageing at 100% $T_{g}$ for 720 h. Notably, this little difference is consistent with the variance of the data.

The decrease of interlaminar shear strength can be attributed to deterioration of the epoxy and of the fibre/epoxy interface. In fact, previous research [11] observed increased void content and fibre/resin debonding in carbon fibre reinforced epoxy, which lead to a reduction of short beam shear strength. Moreover, thermal degradation of the epoxy [17] is thought to contribute to the observed strength decrease.

### 3.4. Effect of Cyclic Ageing

**Figure 4:**
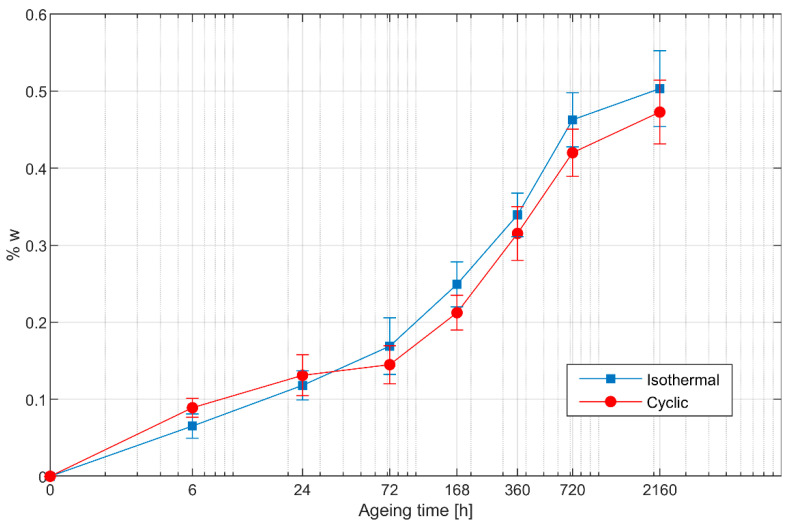
Weight loss for cyclically and isothermally aged specimens. The logarithmic scale on the horizontal axis has been adjusted so that the leftmost point represents the specimens in the unaged condition.

The weight loss caused by cyclic ageing is shown in Figure 4, together with that caused by isothermal ageing at the same value of maximum temperature, i.e., 90 °C. The two ageing conditions produce similar results, with a slightly smaller weight loss in cyclically aged specimens for longer ageing times.

**Figure 5:**
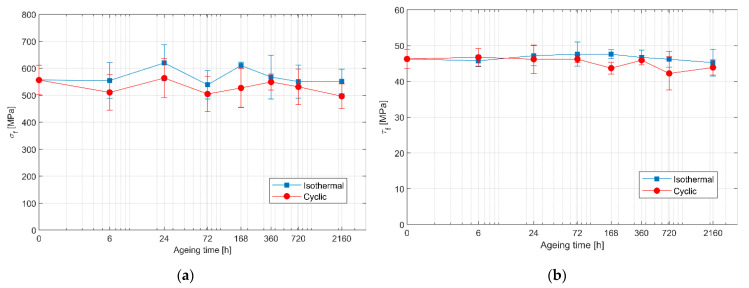
(**a**) Flexural strength and (**b**) interlaminar shear strength of cyclically aged specimens. The results for isothermal ageing at 90 °C are also reported for comparison.

The flexural and interlaminar shear strengths of the specimens subjected to cyclic ageing are reported in Figure 5. Overall, cyclic ageing promotes a greater decrease of flexural strength compared to isothermal ageing, with values that are on average almost 10% lower. On the contrary, the effect on the interlaminar shear strength seems comparable to that of isothermal ageing for short ageing times, while reductions as high as 5% to 10% are found for longer exposure times. For a 6-h duration, the interlaminar shear strength of cyclic ageing seems greater than that of isothermal ageing. However, considering the scatter in the data, the results of the two ageing conditions overlap to a large extent, which would explain this outlier.

It has previously been noted that the effect of isothermal ageing becomes particularly pronounced once the glass transition temperature is reached. Therefore, it is possible that a greater effect of cyclic ageing would also be observed for higher temperatures. Further research, which is beyond the purpose of this work, would be required to investigate more in detail this topic.

## 4. Conclusions

In this work we investigated the effect of thermal ageing on the flexural and interlaminar strengths of carbon fibre/epoxy composites. To this end, several combinations of ageing temperature and time were considered. The mechanical properties of the aged specimens were assessed by three-point bending and short beam shear tests.

Comparing the results of different ageing conditions, we observed that the effect of ageing is mostly dependent on temperature. Isothermal ageing at temperatures below the glass transition temperature ($T_{g}$) resulted in a moderate reduction of flexural strength, with a maximum decrease of about 25% for 2160 h at 75% $T_{g}$. Similarly, a limited decrease of interlaminar shear strength was observed, with a reduction of about 15% for 2160 h at 75% $T_{g}$. Short exposure to sub-$T_{g}$ temperatures could even provide a slight improvement of material strength due to additional post-curing. On the other hand, temperatures equal to or above $T_{g}$ resulted in a rapid decrease of both flexural and interlaminar shear strengths. A 30% strength reduction was observed for ageing times of less than 6 h at 145% $T_{g}$. When designing composite components, it is therefore critically important to ensure that the temperature does not rise beyond critical levels, as even very short exposure to temperatures above $T_{g}$ could result in substantial loss of material strength.

Cyclic ageing was found to affect the mechanical strength in a way similar to isothermal ageing. However, more tests for different temperatures should be conducted to further investigate the response of CFRP to cyclic ageing.

Further research is required to understand the relation between isothermal and non-isothermal ageing, in order to apply the results of accelerated tests to real components. Moreover, future research would be needed to investigate the influence of thermal ageing on the fracture mechanism of CFRP.

## Figures and Tables

**Table 1:** Characteristics of CC 600 ER450 12KT700 pre-preg as reported in the datasheets [28,29].

Material Characteristics	Nominal Value
Fibre diameter [μm]	7
Fibre volume %	51
Fibre tensile modulus [GPa]	230
Fibre tensile strength [MPa]	4900
Fibre strain %	2.1

**Table 2:** Ageing conditions of the CFRP samples. The ageing temperature T, its value expressed as a percentage of the glass transition temperature, and the ageing time in hours are reported.

T	$\%\;T_{g}$	Ageing Time [h]
Room temperature	-	168, 360, 720, 1440, 2160
90	50	6, 24, 72, 168, 360, 720, 2160
135	75	6, 24, 72, 168, 360, 720, 2160
180	100	6, 24, 72, 168, 360, 720
200	110	6, 24, 72, 168
260	145	6, 24, 72, 168

## Data Availability

The data presented in this study are available on request from the corresponding author.
